# Supplementary material for: Validity evidence for assessing social-emotional psychological strengths in Colombian adolescents using the SEHS-S
Source: PLoS One. 2025 Feb 10;20(2):e0314488. doi: 10.1371/journal.pone.0314488 (PMC11809918; doi:10.1371/journal.pone.0314488)
Supplement: Attachment — Submitted filename: Response to Reviewers.pdf [file pone.0314488.s002.pdf]

Response to reviewers

In this document, we address the comments provided by two reviewers on the previous version of the manuscript entitled "Validity evidence for assessing social-emotional psychological strengths in Colombian adolescents using the SEHS-S" (PONE-D-24-25194). We are grateful to both reviewers for their careful reading of this manuscript and their valuable suggestions for improvement. We believe that the revisions we made in response to their feedback have substantially enhanced the quality of this paper.

Note: The line numbers referenced here point to the line numbers in the "Manuscript" document.

Reviewer #1:

Strengths

- *Validity and Robustness of the Model:*
  The hierarchical model of the covitality construct is well-supported through confirmatory factor analyses.
- *Measurement Invariance:*
  Metric and configural invariance have been confirmed across gender and age groups.
- *Cultural Application:*
  The SEHS-S has been validated for the Colombian adolescent population, contributing to the validity evidence in different cultural contexts.
- *Significant Correlations:*
  Moderate to high correlations with related constructs strengthen the convergent validity of the SEHS-S.
- *Large and Diverse Sample:*
  The large sample of 1461 students provides a solid basis for statistical analyses.
- *Practical Implications:*
  The article highlights the utility of the SEHS-S to identify specific needs and propose targeted intervention strategies to improve the socio-emotional well-being of adolescents.

We greatly appreciate this reviewer's recognition of the strengths of our manuscript and are grateful for the precise changes he recommended.

Redundant Parts to Remove and Proposed Modifications

- *Abstract:*
  Lines to remove: 33–34, 35–36
  Remove: "although differences in item functioning across gender and age were found. Furthermore, Covitality maintained moderate to high correlations with related constructs. As a conclusion, the SEHS-S can be considered a valid tool to assess psychological strengths, well-being, and resilience (i.e., Covitality) in Colombian adolescents. However, further research is needed to explore the differences in item functioning."
  Replace with: "though further research is needed to explore item functioning differences across gender and age."

  Reviewer #1 proposes here significantly shortening the summary of results in the abstract, whereas Reviewer #2, in contrast, requests more details, including statistical information (see below). We believe it is important to retain that we found "moderate to high correlations of Covitality with related constructs" (which Reviewer #1 highlighted as one of the strengths of this study). We also think that a paragraph that summarizes the main conclusions of this study is an essential part of the abstract.

- *Introduction:*
  Lines to remove: 47–50 Remove: "Battling these adversities requires the development of strategies that identify emotional and behavioral issues in young individuals and provide support by strengthening their social-emotional growth at early stages and, particularly, with a focus on proactive prevention rather than relying on reactive approaches."
  Replace with: "Developing strategies that identify and address emotional and behavioral issues early, with a focus on proactive prevention, is crucial."

  We have implemented the changes as recommended by the reviewer (lines 54–55).

- *Materials and Methods:*
  Lines to remove: 163–171 Remove: "First, we made the necessary translations and linguistic adjustments to Colombian Spanish for each of the above-mentioned instruments, based on expert judgments and cognitive interviews with Colombian adolescents. Subsequently, the project was presented in the participating schools and after informed consent was obtained from the adolescents and their parents, the four instruments, together with a 15-item sociodemographic questionnaire (asking about age, grade, and sex, amongst others), were applied in groups to the students that were present at school that day. Students were given a brief description of the project and an explanation of the instructions to fill out the questionnaires. The data collection took place in 2019 between March 1 and October 17."
  Replace with: "Translations and linguistic adjustments to Colombian Spanish were made. After obtaining informed consent, the instruments and a sociodemographic questionnaire were administered in groups between March 1 and October 17, 2019."

  We have largely implemented the changes as recommended by the reviewer (lines 179–188). However, as suggested by Reviewer #2 (see below), we included additional information on (a) the adjustments made to the SEHS-S questionnaire for the Colombian context and (b) the logistics of the school visits for the administration of the instruments.

- *Results:*
  Lines to remove: 271–273 Remove: "In interpreting the results in Table 4, recall that the SEHS-S items are scored on a scale from 1 to 4 points and that the intercepts indicate the expected score for an individual with average scores (i.e., equal to 0) on the latent (first-order, second-order, and third-order) factors."
  Replace with: "The SEHS-S items are scored from 1 to 4 points, and the intercepts indicate the expected score for an individual with average latent factor scores."

  We have implemented the changes almost exactly as the reviewer recommends (lines 292–293).

- *We invite you to consult the article by Diotaiuti et al. (2017), "Alcohol drinking patterns in young people: A survey-based study" published in the Journal of Health Psychology, for a detailed analysis of youth behaviors that could enrich the discussion on the importance of psychological strengths and socio-emotional behaviors in adolescents. You can insert this citation in the Introduction section, in the paragraph discussing the importance of understanding the psychological and socio-emotional behaviors of adolescents.*

  Example of Insertion:
  "Understanding the behavioral patterns and psychological strengths of adolescents is crucial for developing effective interventions. We invite you to consult the article by Diotaiuti et al. (2017) for a detailed analysis of youth behaviors that could enrich this discussion (Diotaiuti, 2017)."

  As suggested by this reviewer, we have now cited in our introduction (lines 64–68) the 2017 paper published in the *Journal of Health Psychology*, titled "Alcohol drinking patterns in young people: A survey-based study" of which Martinotti is the first author (and Diotaiuti is one of the co-authors). This paper illustrates how focusing on adolescents' psychological strengths can be beneficial for developing effective interventions.

Reviewer #2:

This paper presents a comprehensive overview of the validity evidence obtained through the SEHS-S for the Common Vitality Assessment for Colombian adolescents, which has some theoretical value and practical implications. However, there are some problems with this paper that require revision by the authors.

We thank Reviewer #2 for his/her constructive feedback on the previous version of our manuscript. We hope that the revised version adequately addresses the issues that were raised.

Detailed information:

- *Abstract:*
    1) *Abstract requires a brief overview of the background, purpose, methods, results, and conclusions of the article. It is recommended to add subheadings to make the structure of the article clearer.*

       In the revised version, we have structured the abstract along the lines suggested by this reviewer, providing a brief overview of the main elements in each section.

    2) *The results section of the abstract is to be narrated with relevant statistical data, and it is recommended that the results section be rewritten with reference to high quality literature.*

       The Results section of the abstract now includes key statistics (particularly regarding the goodness of fit of the hierarchical factor model for the Covitality construct measured by the SEHS-S, which is the main finding of the current study; lines 35–37). To keep the Results section of the abstract concise (as requested by Reviewer #1, see above), we have excluded detailed statistical information related to the results for secondary analyses, such as those for measurement invariance.

       We are unsure if we fully understand this reviewer's recommendation that "the results section be rewritten with reference to high quality literature". PLOS ONE explicitly states that the abstract should not include any references. Does this reviewer suggest adding references to the manuscript's Results section? This also seems unusual as the Results section typically presents the study's findings without relating them to other studies (such reflections are usually reserved for the Discussion section). If the reviewer could clarify what is exactly meant by "the results section be rewritten with reference to high quality literature" or provide specific suggestions for references to include, we would be happy to make the necessary revisions.

- *Materials and methods — Participants*
    - *Line 104–105, page 5: "four public and rural secondary schools"*
      How were these middle schools chosen? Why were these four middle schools chosen? Can you tell us the exact process.

      We have now added (lines 114–116) that the four participating schools were selected for convenience and because they are the most important secondary schools in the department of Risaralda (where the university is located to which the three Colombian authors of this manuscript had their primary or secondary affiliation).

    - *Overall:*
        1) *Is the 1461 sample size the final sample size?*

           Yes, 1,461 is the final size used for all data analyses in this manuscript. We have clarified (lines 116–117) that all participants met the inclusion criteria, ensuring that the final sample consists of all 1,461 students who volunteered to participate.

2) *1461 Is the sample size sufficient? What formula was used to calculate the minimum sample size?*

   Before the data collection, we anticipated that more than 1,000 students would participate based on the size of the participating schools. Literature recommendations as well as common practices (with similar validation studies involving the Covitality construct; e.g., Piqueras et al., 2019), suggest that this sample size is sufficient for the planned analyses. We have also added (lines 118–120) two references to support this statement.

3) *During the questionnaire collection process, were there any missing rates and what were they? What was done about the missing data.*

   Already in the previous version of the manuscript, we noted that the nonresponse rate, across all instruments was relatively low (1.0%; lines 247–248) and that, to address the missing data problem in the analyses, which typically require complete records for the individuals, we employed multiple imputation (lines 196–198).

4) *Are there inclusion and exclusion criteria for the population? If so, please specify in the article.*

   We have added (lines 116–118) that the inclusion criteria required a minimal level of cognitive ability and reading skills to complete the self-report questionnaires.

- *Procedure*
  - *Line 164–166, page 8: "necessary translations and linguistic adjustments to Colombian Spanish for each of the above-mentioned instruments"*
    Can you be more specific about the process?

    We have provided additional information on the adjustments we made to the SEHS-S based on expert evaluations and cognitive interviews (lines 179–182).

  - *Line 169–170, page 8: "were applied in groups to the students that were present at school that day"*
    Is it all the students who were in school that day? Or was the sampling done through some sort of sampling method? Please elaborate.

    We now explicitly state (lines 186–188) that we administered the instruments to all students from a specific grade who were present at school that day and had provided their consent.

  - *Line 171–172, page 8: "The data collection took place in 2019 between March 1 and Octobre 17."*
    Can you state the collection time for each school separately?

    As follows from our response to the previous comment, each school was visited multiple times to administer the instruments to specific grade levels. We have clarified this on lines 185–186.

- *Discussion*
  1) *What are the limitations of this paper?*

     We have added at the end of the discussion section (lines 423–432) a paragraph with the limitation of the study.

- *Conclusions*
  1) *It is suggested that the conclusions be shortened.*

     We have shortened the Conclusions section by more than one-third (from 155 to 96 words).
