# Supplementary material for: Validity evidence for assessing social-emotional psychological strengths in Colombian adolescents using the SEHS-S
Source: PLoS One. 2025 Feb 10;20(2):e0314488. doi: 10.1371/journal.pone.0314488 (PMC11809918; doi:10.1371/journal.pone.0314488)
Supplement: Attachment — Submitted filename: Response to Reviewers.pdf [file pone.0314488.s003.pdf]

Response to reviewers

In this document, we address the comments provided by two reviewers on the previous version of the manuscript entitled “Validity evidence for assessing social-emotional psychological strengths in Colombian adolescents using the SEHS-S” (PONE-D-24-25194R1). We are grateful to both reviewers for their careful reading of this manuscript and their valuable suggestions for improvement. We believe that the revisions we made in response to their feedback have substantially enhanced the quality of this paper.

Note: The line numbers referenced here point to the line numbers in the “Manuscript” document.

Reviewer #1:

General comment

Thank you very much for the opportunity to review this well-conducted study. I particularly appreciated the innovative approach and the detailed attention you demonstrated in assessing the socio-emotional psychological strengths of Colombian adolescents through the Covitality model. It is clear that you have put great effort into producing a rigorous and culturally meaningful research. My heartfelt congratulations to all the authors for the excellent quality of the work.

The manuscript presents several noteworthy aspects that deserve to be highlighted:

- *Solid theoretical approach: The description of the Covitality model and its application in different contexts is clearly presented, highlighting the broad scope of the construct and its relevance in the field of mental health.*
- *Rigorous statistical methodology: The use of confirmatory factor analyses (CFA) and the verification of gender and age invariance are effective tools for assessing the validity of the scale used.*
- *Contribution to local literature: The validation of SEHS-S in the Colombian context is a valuable contribution to positive psychology in Latin America, where similar instruments are scarce.*
- *Balanced discussion: The reflection on the results, particularly on gender and age differences, is well-founded and provides an important basis for future targeted interventions*

We greatly appreciate the reviewer’s positive feedback on our manuscript and are thankful for the suggested changes.

Suggested revisions

- *Abstract*
  - *Line 35: Add numerical values for the correlations to improve clarity. Example: “Covitality maintained moderate (r = 0.5) to high (r = 0.7) correlations with related constructs.”*

    We have added the range of the correlations between the SEHS-S and the other instruments (Kidscreen-10, MHI-5, TEIQue-ASF, and PSC-17-Y) [line 40].

- *Introduction*
  - *Line 47: I suggest expanding the description of prevention strategies with concrete examples of interventions based on socio-emotional strengths, such as school or community programs.*

    We have added a paragraph to elaborate on prevention strategies based on covitality and socio-emotional strengths [lines 82–96].

  - *Line 61: The phrase “psychological anxiety” could be more specific, clarifying whether it refers to specific anxiety disorders or a more general condition.*

    We have changed the term “psychological anxiety” to “emotional distress”, hoping this clarifies that we are referring to a more general condition [line 79].

- *Materials and Methods*
  - *Line 104: Add a brief note on the criteria for selecting the participating schools to improve the transparency of the sampling. Example: "Schools were selected based on their geographic and socioeconomic representativeness within the department of Risaralda."*

    We are unsure on how to handle this comment. As we stated in the previous version [lines 129–131, "These schools were selected by convenience, for their proximity, accessibility, and for being the largest schools in the department"; lines 129–131 in the current version of the manuscript], the schools were selected by convenience rather than for their representativeness in Risaralda. While these are the largest schools in the department, allowing us to obtain a large and relatively representative sample for Risaralda, we cannot fully guarantee the latter.

  - *Line 172: Correct "Octobre" to "October."*

    We corrected this typo [line 203].

  - *Line 183: Providing a brief justification for using SAS V9.4 could help clarify the software choice. Example: "SAS V9.4 was chosen for its robustness in multivariate data analysis and its ability to handle large datasets with missing values."*

    We have added a sentence to clarify the specified properties of SAS Software [lines 241–242].

- *Results*
  - *Line 226: Quantify the statement "relatively high nonresponse rate" for greater clarity. Example: "The nonresponse rate was 22.7% for the TEIQue-ASF, the highest among the instruments used."*

    We have added the nonresponse rate (percentage of participants with at least one missing response) for the TEIQUE-ASF (with detailed information presented in Table 2) [line 270].

  - *Lines 225–227: Briefly discuss the potential impact of the nonresponse rate on the study's conclusions. Example: "Although the overall nonresponse rate was low, its concentration in longer instruments may have influenced the results, particularly for the SEHS-S."*

    We have added the phrase "As explained in the Data Analysis section, missing responses were imputed under the missing-at-random assumption. Given this assumption and the relatively low nonresponse rate at the item level, their impact on the results and conclusions is expected to be minimal" [lines 270–273]. Although there are a somewhat large number of participants with one or a few missing responses (e.g., 14.1% on the SEHS-S), the overall small number of missing responses (on the item level; 0.6% for the SEHS-S) makes it unlikely that the missing data have significantly influenced the results and conclusions.

- *Discussion*
  - *Line 333: Expand the comparison with similar studies in other cultural contexts. Example: "These findings are consistent with those obtained in studies conducted in Spain and Japan, where the hierarchical structure of the Covitality model has been confirmed, albeit with slight variations in the lower-order factors."*

    We are uncertain about what the reviewer precisely means with "albeit with slight variations in the lower-order factors". In fact, the hierarchical structure of the Covitality $1 \to 4 \to 12 \to 36$ model in our paper is *identical* to the model employed in other cultures. There are no differences in the structure of either the 12 lower-order or 4 higher-order factors (nor in the 36 individual items). This indicates that configural invariance across cultures has been established. We have added the latter clarification to the Discussion [lines 394–395].

– *Line 370: Replace "further investigated in future research" with a more specific phrase. Example: "Further investigations, particularly longitudinal studies, will be needed to explore how gender and age differences evolve over time."*

We have made the adjustment as suggested by the reviewer [lines 447–448].

Limitations

Despite the considerable value of this work, there are some limitations that should be addressed to ensure greater transparency and completeness of the manuscript:

- *Self-report bias: Given the self-report nature of the instruments used (SEHS-S and other questionnaires), there is a potential risk of bias related to social desirability or the adolescents' ability to accurately assess their own emotional state. This aspect should be acknowledged and discussed, as it may affect the results, especially in the measurement of subjective well-being.*

  We have added this limitation in the new subsection "Limitations" (within the Discussion section) [lines 457–464].

- *Limited sample representativeness: Although the sample is large, it focuses mainly on adolescents from rural and socioeconomically disadvantaged backgrounds in a specific region of Colombia. This may limit the generalizability of the results to other geographical areas or adolescents from different socioeconomic groups. Mentioning this limitation would help clarify the boundaries of the study's applicability.*

  We believe this limitation was addressed in the previous version of the manuscript [lines 423-–428 of the previous version; lines 451—456 of the current version]. Specifically, we explicitly noted that any generalization beyond the current sample—both in time and space—is highly speculative.

- *Lack of longitudinal data: The study provides a cross-sectional snapshot of SEHS-S validity. However, it would be interesting to explore how socio-emotional psychological strengths develop over time in adolescents. Longitudinal studies could clarify the evolution of the gender and age differences found.*

  We have not explicitly included this limitation in the new Limitations subsection, as our study did not aim at exploring how covitality evolves over time (potentially differing between boys and girls). However, as mentioned above, we did note that future studies employing a longitudinal design may shed a light on this development.

- *Unresolved scalar invariance: The lack of scalar invariance in some SEHS-S items between gender and age groups limits the ability to make direct comparisons between these groups. Although this has been discussed, further emphasis on how this impacts data interpretation would be beneficial.*

  We view the finding that scalar invariance does not hold in our sample not as a limitation of our study but rather as a limitation of the SEHS-S itself, which, as the reviewer correctly pointed out, should be considered when comparing groups of different genders and/or ages. We believe we have thoroughly discussed the implications of this finding in other sections of the manuscript.

Sections to Eliminate

There are no sections that need complete elimination, but some parts could be streamlined for greater clarity. For example:

- *The sections between lines 330–350 on the confirmation of the factor model could be condensed to avoid repetition while keeping the essential points.*

  We are unclear about the reference to lines 330–350 in the previous version of the manuscript (which the reviewer evaluated). These lines include the last sentences of the "Factorial Structure and Invariance" subsection (lines 330–333), the "Gender and Age Differences" subsection (lines 334–346), and the beginning of the Discussion section (lines 347–350). (Also the Revised Manuscript with Track Changes does not address the "confirmation of the factor model" in lines 330–350; rather, it focuses on factorial invariance.) Furthermore, the section discussing the confirmation of the factor model (lines 366–370 in the previous version of the manuscript; lines 390–395 in the revised manuscript) contains only six lines of text. If the reviewer could specify precisely which part of the text he would like us to rewrite in a more condensed way, we would be happy to make the adjustments.

Extra citation

I suggest that the authors cite "A Structural Model of Self-Efficacy in Handball Referees" by Pierluigi Diotaiuti and colleagues, published in Frontiers in Psychology (2017). This article focuses on the role of self-efficacy in sports contexts and could be relevant to your study on psychological strengths in adolescents, particularly when discussing the importance of self-belief and emotional competence as factors contributing to resilience and well-being. The framework of self-efficacy in the referenced article aligns with themes of personal psychological strengths that could enrich your discussion.

In the Introduction, after discussing the Covitality model and its components (lines 56-58), you could introduce this citation when expanding on the significance of self-efficacy as a psychological strength that promotes resilience in various contexts.

Example of Citation:

You could say: "The importance of self-efficacy in fostering psychological resilience has been demonstrated across different populations, including in sports contexts, where it has been shown to significantly impact performance and coping mechanisms (Diotaiuti et al., 2017)."

This will strengthen the theoretical framework of your study by linking it to relevant research on psychological strengths in non-clinical settings.

We thank the reviewer for bringing the article by Diotaiuti et al. (2017) to our attention. While we appreciate his suggestion, we believe that this article, although interesting, is not directly relevant to the main focus of our study. Our research primarily aims at covitality and although the concept of self-efficacy is certainly relevant for covitality —it is one of the twelve first-order factors in the Covitality $1 \to 4 \to 12 \to 36$ model— we believe that the specific findings of Diotaiuti et al. do not significantly contribute to our current arguments. (If we included the paper by Diotaiuti et al., we should include also one or more papers for each of the other first- or second-order constructs in the Covitality model.) Therefore, although we carefully considered the reviewer's suggestion, we have decided to maintain our current bibliography.

Reviewer #2:

As a result of the author's revisions, the article has become progressively standardized and acceptable, however, there are still some minor problems, so the author is requested to revise it carefully.

We thank Reviewer #2 for his/her constructive feedback on the previous version of our manuscript. We hope that the revised version adequately addresses the issues that were raised.

Materials and methods

1) *"The inclusion criteria, which were met by all participants, required a minimal level of cognitive ability and reading skills to complete the self-report questionnaires." What were the inclusion and exclusion criteria for participants and please specify. How can it be demonstrated that the participant has a minimum level of cognitive and reading ability to complete the questionnaire?*

   We have added more details in the Participants subsection now. We have specified that "to be eligible for participation, students had to demonstrate basic reading and writing skills and be free from any diagnosed mental or cognitive disabilities" and that "these inclusion and exclusion criteria were verified by both the students' teachers and the psychologists at the participating schools" [lines 131-134].

2) *What is the total sample size for the final fill? How many of these were valid? Were there any invalid questionnaires in this survey? How were invalid questionnaires handled?*

   We have now more precisely added that the original sample included 1,473 participants but that 12 participants were excluded for further analyses because of a large amount of nonresponse. [lines 135–138]
